# Optimizing Reaction Time in Relation to Manual and Foot Laterality in Children Using the Fitlight Technological Systems

**DOI:** 10.3390/s22228785

**Published:** 2022-11-14

**Authors:** Dana Badau, Adela Badau

**Affiliations:** 1 Petru Maior Faculty of Sciences and Letters, George Emil Palade University of Medicine, Pharmacy, Sciences and Technology, 540142 Targu Mures, Romania; 2 Interdisciplinary Doctoral School, Transilvania University of Brasov, 500036 Brasov, Romania

**Keywords:** reaction time, hand laterality, food laterality, Fitlight technology, male and female, children, physical education, right and left motor prevalence’s

## Abstract

The purpose of the study was to design and implement, in the physical and sports education process and in the motor evaluation process, a program of exercises and specific tests to optimize reaction time by using the Fitlight technological systems in relation to the manual and foot laterality of the pupils and identification of gender differences regarding the development of reaction speed. The study included 231 pupils, between 10 and 11 years old, who were divided into two groups according to gender, as follows: the male sample included 109 (97.32%) subjects, and the female sample included 103 (94.45%) participants. All subjects were identified with right manual and foot laterality. Both samples performed a specific exercise program to optimize reaction time in relation to manual and foot laterality by using Fitlight technologies. In the study, four tests were applied in order to evaluate reaction times using Fitlight, two in relation to the manual laterality and two with foot laterality, and the results were statistically processed with IBM SPPS Statistic 24 (IBM Corp., Armonk, NY, USA). Through the comparative analysis of the samples and the progress aimed at optimizing the reaction time specific to our study, it was found that the female sample recorded greater progress at the level of manual laterality, both for the right hand and for the left one, while the sample of boys recorded significant progress in terms of improving reaction time at the level of right and left foot laterality. At the foot laterality level, the results for the executions with the right foot were better in the simple test with four Fitlight spotlights in a line, and for the complex test, with eight Fitlight spotlights in a square, the results were better in the executions with the left foot. This reveals the fact that the greater the execution complexity, the better the motor prevalence on the left side.

## 1. Introduction

The optimization of motor capacity development is conditioned by human physical and mental transformations in order to form complex and adaptive behaviors to the natural and social environment. The set of motor aptitudes that an individual can possess is unlimited, due to the multiple possibilities of combining movements and of their adaptation to a varied series of factors and varied situations. Seen from the outside, human motor behavior appears as a complex chain of movements, attitudes, or postures through which humans adapt to different, ever-changing conditions of the environment [1,2,3].

The process of developing the psychomotor capacity of pupils is a major concern of specialists in the field of physical activities. Studies on the development of psychomotricity are numerous and have mainly focused on its components: reaction time (RT), coordination, body scheme, on manual or foot laterality, and on ideomotricity [4,5,6,7,8]. Recent research has been aimed at identifying the intercorrelation between the components of psychomotricity, with a focus on identifying correlations between laterality and human reactions, between spatial orientation and reaction speed and coordination, and on interaction between cognitive and motor reactions [9,10,11].

Psychomotricity, an important component of the complex psychobehavioral system, develops through a complex process that facilitates the interference of mental factors with motor factors and which is majorly influenced by the content and diversity of the physical training program, and by human predispositions and the plasticity of the central nervous system [12,13,14]. Studies demonstrate that the most favorable age for the development of psychomotor components is between 1 and 14 years, being conditioned by the complexity of the systematically and scientifically directed physical exercises carried out [15,16,17,18].

The reaction time (RT) represents an important component of psychomotricity which is influenced by: the genetic peculiarities related to the speed of transmission of the nervous influx to the synapses and concretized in the motor effector response, by temperamental aspects, and by the motor experience of the subject [19,20,21]. The motor reactions of the practitioners of physical and sports activities are elaborated responses to certain stimulations. In physical activity, knowing and mastering the methodology of using the most appropriate stimuli embodied in physical exercises are the prerequisites for optimizing motor behavior.

A series of studies have concluded that the FitLight Trainer™ System [22] can be used as a training and evaluation tool with a high level of reliability, validity, versatility and portability in measuring simple and complex reactive time to visual stimuli in different categories of healthy populations of athletes and non-athletes [23,24,25]. The Fitlight Training System was used to study reactions to visual stimuli in physical education lessons, demonstrating that the use of these devices contributes to improving simple and complex reaction time in relation to the selective attention of primary school students [26,27]. Another extended application of the Fitlight system targeted performance sports where it was used to train and measure reaction times to visuomotor tasks at the level of: manual laterality, foot laterality, hand-eye, and foot-eye coordination. The studies concluded that the use of these devices is simple, valid, and reliable, and contributes significantly to the optimization of the athletes’ reaction times [28,29,30,31,32,33]. The implementation of these devices in physical education lessons facilitates the performance of exercises with a large number of students. The costs of the devices are relatively acceptable, the portability of the devices is high, and the layout and configuration of programs with Fitlights allows for diversified options that can be adapted to individual particularities and to the subjects’ motor potential development targets.

The specific functional asymmetry of the structure of the cerebral hemispheres is defined from the point of view of the motor prevalence of the left or right manual or foot laterality, on the basis of which the subjects are classified as: right-handed, left-handed, ambidextrous, or derivatives of these, such as those with crossed laterality [34,35,36,37]. In the execution of motor actions, the cerebral hemispheres participate unequally, depending on the nature of the task being performed, but it is very important to note that in carrying out a motor task, most of the time, the two cerebral hemispheres complement each other and interrelate [38,39]. One of the problems of scientific research in the field of laterality is the question of whether functional asymmetry is an effect of genetic conditioned maturation or only an educational effect conditioned by social, cultural and environmental factors [40,41]. We consider that both theories are correct; laterality is conditioned both by genetic factors, as well as by educational, social, cultural and environmental factors. The process of physical and sports training aims at a balanced development of the upper limbs, the lower limbs, and, implicitly, the two sides of the body: left and right [42,43,44,45].

The development and implementation of information technologies in the field of motor activities currently occupies a relevant place in the training process specific to physical and sports education and motor evaluation. We consider that the exercises that use information technology are recommended in order to develop laterality and, implicitly, the symmetrization of reactions and human motor skills. Studies at the level of children which highlight that the reaction time (RT) in performing different motor actions to visual or auditory stimuli is conditioned by laterality are relatively common, and analyze these reactions mainly from the perspective of age or motor and cognitive level [46,47,48,49], but fewer studies take into account the perspective of gender differences and manual and foot laterality. Understanding the defining aspects and differences in the manifestation of motor reactions in relation to laterality depending on gender, we consider that our study will contribute to expanding knowledge, and understanding the interrelationship between psychomotor and biological development. The studies that looked at the differences between manual and foot laterality in students using the Fitlight system are relatively few, and they focus predominantly on improving reaction times to visual stimuli and not on identifying laterality differences and gender differences in the manifestation of reaction time. The novelty of our study consists in the design and implementation of a physical exercise program that uses Fitlight technology in the process of improving reaction time in relation to the development of manual and foot laterality of secondary school students within lessons of physical education and sports. We also designed and tested a set of assessment tools (four tests, two simple and two complex) of reaction time and manual and foot laterality using Fitlight technology.

The main purpose of this study is aimed at the conception and implementation, within the physical and sports education process and in motor evaluation, of a program of exercises and specific tests to optimize the reaction time by using the Fitlight technological systems in relation to the manual and foot laterality of the pupils. Moreover, the second goal is aimed at identifying gender differences in terms of the development of reaction speed in relation to manual and foot laterality by implementing a physical exercise program that uses Fitlight technology.

## 2. Materials and Methods

### 2.1. Participants

Inclusion of subjects in the experiment was based on informal consent regarding the purpose of the study and the work schedule. Included in the study were 231 pupils, aged between 10 and 11 years old, who were divided into two groups according to gender as follows: the male experimental group and the female experimental group. Both samples followed the same experimental program for the development of reaction time and manual and foot laterality. The male sample included 109 (97.32%) subjects with right manual and foot laterality, and the female sample included 103 (94.45%) subjects with right manual and foot laterality. Inclusion criteria: students aged between 10 and 11 years, right manual and foot laterality, active and healthy pupils, and the complete performance of the reaction time optimization program and the evaluation tests proposed for this research. The manual laterality was identified by the action of throwing the tennis ball with the hand towards a fixed point, and the foot laterality by the preference of hitting the soccer ball placed on the ground. Written informed consent to participate in this study was provided by the participants’ parents or legal guardians.

### 2.2. Study Design

During the research, the Fitlight system was used for training and evaluation of reaction time. The study was designed and structured in relation to the intended purpose of training and evaluating children’s reaction times, which represents an objective of physical education at the primary education level, and the use of sensor-based technologies facilitates increasing the attractiveness of lessons and providing accurate results in real time. The Fitlight^®^ System is designed as a dynamic system especially for training and assessment of motor ability [50]. We selected the Fitlight Training System for this study for the following reasons: it is validated, reliable, portable, simple to use, and offers real-time feedback. The system is composed of wireless LED spots. Fitlight^®^ is marketed as a patented, fully programmable sensor with tactile impact (when touching spikes) and kinetic impact (when performing movements). The spikes have very high durability, being flexible and with multiple possibilities of arrangement and integration. The spikes (lights) have a range of 50 M–75 M calculated from system to wireless system. The spikes are completely portable, and the software contains 30 preset training programs, but with multiple customization possibilities. Packages are available from 4 to 32 spots. As part of our research, we had 2 packages of 8 spotlights, each with default software. The results of the software can also be downloaded and viewed through applications on mobile phones or laptops. The software provides timely results so that training and testing programs are reliable and allow real monitoring and optimization of the targeted motor factors and components.

Fitlight has multiple uses, aimed at: speed development; development of coordination; developing reaction and response time; cognitive processing function training; development of spatial awareness and orientation; improving fine motor control; and development of peripheral vision.

Research phasing:

- initial testing: 26–30 September 2021;
- implementation of the exercise program (20 lessons) 5 October 2021–11 December 2021;
- final testing: 14–18 December 2021.

### 2.3. Experimental Program of Study

The exercises within the experimental program were designed in such a way as to use the Fitlight device and were specially adapted to the age and motor skills of the subjects in the female and male experimental groups. The program was identical for the female and male samples. We mention that the education system in Romania provides for 2 lessons of 50 min per week of physical and sports education for the primary and secondary levels.

**Figure 1:**
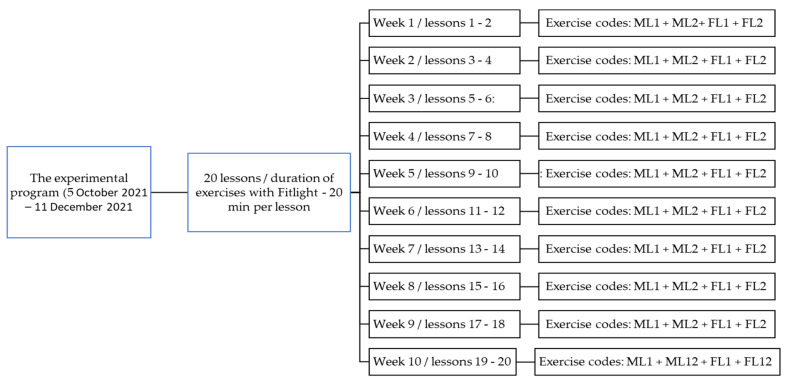
Scheduling of exercises per lesson during the experimental program.

In order to assign the exercises to the lessons within the experimental period, we coded each exercise as follows: exercises for the development of reaction time in relation to the education of manual laterality received the ML code, being numbered ML1–ML12; exercises for the development of reaction time in relation to the education of foot laterality received the code FL, being numbered FL1–FL12. The sequence of these exercises for each lesson is presented in Figure 1.

Examples of exercises:

- ML2: standing 1 m away from a gymnastics bench where 2 spots are placed at a distance of 2 m between them, moving to the gymnastics bench and touching the blue spot with the right hand or the white spot with the left hand and returning to the line of departure; the number of successes for each hand per 30 s is quantified;
- ML4: dissonance of 6 spotlights randomly on a square panel with a side of 1 m, the student must touch with either hand as many of the light spots as possible in 30 s;
- ML5: similar to exercise ML4, but the blue spots will be touched with the right hand, and the white spots with the left hand; the number of successes for each hand per 30 s is quantified.
- FL3: a triangle ABC is drawn on the ground, the student sits in corner A, and 4 spots are placed on the side BC with a length of 2 m; the student must move and touch as many of the light spots as possible with either foot; the number of successes in 30 s is quantified;
- FL7: a circle with a diameter of 2 m is drawn on the ground, and 8 spots are placed on its circumference; the student must identify the light spot and touch it with either foot, the number of successes in 30 s is quantified;
- FL10: two parallel lines with a length of 2 m are drawn on the ground, 2 m apart; 4 spots are placed on each line, and the spots light up randomly; the student is placed in the center, at the end of the two lines, and the student must touch with his right foot the light spots on the right line and with his left foot the light spots on the left line; the number of successes for each leg per 30 s is quantified.

Manual and foot laterality is conditioned by the laterality of the eyes (ocular laterality), which together influence movement performance and motor behavior. In our study, the exercises from the experimental program, as well as the performance of the motor tests, were performed with the visual control of both eyes, there being no restrictions for the involvement of only one dominant eye.

### 2.4. Measures

The research included four tests for the evaluation of reaction time and laterality, as follows:

- the Fitlight test 4 spots in line—manual execution (right hand/left hand);
- the Fitlight test 4 spots in line—foot execution (right leg/left leg);
- the Fitlight test 8 spots in a square—manual execution;
- the Fitlight test 8 spots in the square—foot execution.

The Fitlight test 4 spotlights in line—manual execution (right/left) aims to evaluate hand-eye coordination and reaction speed. The seated pupil stands in front of a table with a height of 80 cm. On the table, 4 spotlights are placed in a line at a distance of 30 cm between them. The pupil must touch with the palm or the fingers of the hand as many spotlights as possible that illuminate during 30 s. For each hand, the best performance recorded from 2 trials was recorded. The Fitlight test 4 spotlights in a line—foot execution (right/left) follows the evaluation of eye-foot coordination and reaction speed. The student in the stand is placed in front of one line at a distance of 30 cm. On the soil, 4 spots are placed in a line with a distance of 30 cm between them. The student must touch as many spotlights as possible with the ball of the foot during 30 s. For each leg, the best performance recorded from 2 trials was recorded.

The Fitlight test 8 spotlights in a square—manual execution consists of evaluating hand-eye coordination and reaction speed. The pupil stands in the middle of a square with a side of 2 m. The 8 spotlights are arranged at a distance of 100 cm between them. The student must touch with the palm or the fingers of the hand as many spotlights as possible that illuminate during 30 s. The best performance recorded from 2 trials was recorded. The lighting order of the spotlights is random, and touching the spotlights can be performed with either hand.

The Fitlight test 8 spotlights in a square—foot execution evaluates eye-foot coordination and reaction speed. The pupil stands in the middle of a square with a side of 2 m. The 8 spotlights are arranged with a distance of 100 cm between them. The student must touch with the tip of the foot as many spotlights as possible that light up for 30 s. The best performance recorded from 2 trials was recorded. The lighting order of the spotlights is random, and touching the spotlights can be performed with either of the two feet.

### 2.5. Statistical Analysis

The results of the study were analyzed with IBM SPSS 24 software (IBM Corp., Armonk, NY, USA). The following parameters were used in this research: average (X), standard deviation (SD), and average difference between tests (DX). In addition, for the comparative analysis of the progress and differences between the female and male samples, we used the following statistical parameters: the Student’s *t*-test (*t*), the confidence interval with two levels, lower and upper (95% CI), and d effect size. The intervals of interpretation of the Cohen’s d effect size were: 0.1–0.2 small, 0.3–0.5 medium, 0.5–0.8 large, and over 0.8 very large. For the present study, the reference value for the interpretation of statistical significance was *p* < 0.05. To calculate the reliability of the Fitlight System, we calculated the intraclass correlation coefficient (ICC) and standard error of measurement (SEM). For the interpretation of the ICC, we used the following intervals of reliability: values below 0.5 poor reliability, between 0.5 and 0.75 moderate, between 0.75 and 0.9 good and any value above 0.9 indicates excellent [51].

## 3. Results

The results of the manual reaction time in the Fitlight test 4 spotlights in a line (Table 1) highlight better results in the executions with the right hand as compared to the left, both at the initial and at the final testing. In Table 1, it can be seen that the female sample recorded superior progress compared to the male sample, both for the right manual executions of 0.334 executions, and for the left hand of 0.152 executions. The progress registered by the female sample was on average 1.359 executions for the right hand and 0.756 executions for the left hand, the difference between the two segments being 0.755 executions in favor of the right hand; the male sample registered a progress of 1.025 executions for the right hand and 0.604 executions for the left hand, the difference between manual segments being 0.269 executions in favor of the right hand. The values of the arithmetic mean differences recorded between the two samples fell between the two lower and upper limits of the confidence coefficient 95% CI. The average progress recorded by both samples was statistically significant for *p* < 0.05. The analysis of Cohen’s parameters reflects a large effect size for both samples, both for right-handed and left-handed performances, with the recorded values falling between 0.520 and 0.760. The results showed for the right hand ICC = −0.872, SEM = 0.134 for the female group and ICC = 0.715, SEM = 0.183 for the male group, values that reflect adequate reliability. The results for the left hand show that ICC = 0.712, SEM = 0.207 for the female group and ICC = 0.702, SEM = 0.193 for the male group, and the results fall within the good limits of the reliability index.

The results in Table 2 show that the male sample recorded superior progress compared to the female sample, both for the right and left leg executions. The progress recorded by the executions with the left foot were superior to those of the test with the right foot. The female sample recorded a progress of 0.819 executions with the right leg and 1.329 executions with the left one; the average differences for both recorded legs were statistically significant and fell between the lower and upper limits of the confidence coefficient 95% CI. The application of the experimental program to the female sample revealed an effect size of 1.625 for the test performed with the right leg and of 0.546 for the one with the left leg, values that reflect an average effect size. The male sample recorded a progress of 1625 executions with the right leg and 11,607 executions with the left, the average differences for both recorded legs were statistically significant and fell between the lower and upper limits of the confidence coefficient 95% CI. The application of the experimental program to the female sample revealed an effect size of 0.685 for the right leg test and 0.607 for the left leg, values that reflect a large effect size. The reliability results of the Fitlight test 4 spotlights in line—foot execution were for the right leg ICC = −0.781, SEM = 0.172 for the female group and ICC = 0.821, SEM = 0.187 for the male group; and for the left leg, ICC = −0.834, SEM = 0.215 for the female group and ICC = 0.861, SEM = 0.211 for the male group, results that highlight good reliability. Analyzing the two samples comparatively, in the reaction time test (RT) Fitlight test 4 spotlights in a line—right leg execution, there was a difference of 0.806 executions in favor of the male sample, and for the left foot executions of 0.278 executions also in favor of the sample male.

In the Fitlight test 8 spotlights in a square—manual execution, it can be seen that the greatest progress regarding the reaction speed recorded was for the female sample in the right-handed executions of 1183 executions, and the lowest was recorded by the male sample in the left-handed executions of 0.871 (Table 3). The female sample recorded greater progress compared to the male one, in both executions, so for the right hand the differences were 0.103 executions, and for the left hand 0.207 executions. The differences recorded between the tests, as a result of the application of the experimental program, were statistically significant for *p* < 0.05 and fell between the 95% CI limits. For both samples, the effect size was large for the right-hand test d > 0.5 and medium for the left-hand tests d < 0.5. For the right hand, the results were ICC = −0.812, SEM = 0.316 for the female group and ICC = 0.834, SEM = 0.213; for the left hand, ICC = 0.782, SEM = 0.221 was recorded for the female group and ICC = 0.795, SEM = 0.151 for the male group, the results that fall within the good limits of the reliability index.

Table 4 shows the results of the Fitlight test 8 spotlights in a square—leg execution, where it can be observed that the male sample recorded superior progress compared to the female sample, both for the right leg executions of 0.628 executions, and for the left leg of 0.427 executions. The progress registered among the female test subjects was 0.750 executions for the right leg and 0.659 executions for the left leg, the difference between the two segments being 0.091 executions. The differences recorded between the arithmetic averages of the final and initial tests were statistically significant and fell within the 95% CI limits, and the effect size for both higher segments fell within the average limits. The progress registered between the test subjects of the male sample was 1.216 executions for the right leg and 1.086 executions for the left leg, the difference between the two segments being 0.130 executions. According to Table 4, the differences recorded between the arithmetic averages of the final and initial tests of the male sample were statistically significant for *p* < 0.05. The values of the recorded progress values fell between the lower and upper limits for the 95% CI; the size of the effect was 0.683 for the right leg and 0.580 for the left leg, values that fall within the limits considered wide. The reliability of the Fitlight test 8 spotlights in a square—foot execution was good, thus for the right leg, the results were ICC = −0.809, SEM = 0.274 for the female group and ICC = 0.728, SEM = 0.1843 for the female group males, and for standing leg executions ICC = 0.773, SEM = 0.314 was recorded for the female group and ICC = 0.712, SEM = 0.192.

## 4. Discussion

The present study focused on the optimization of reaction time in relation to manual and foot laterality by implementing a specific program that uses Fitlight technology. We also aimed to identify gender differences (male versus female) regarding reaction time in relation to manual and foot laterality by implementing an exercise program that uses Fitlight technology. We consider that the results of our study will facilitate the expansion of knowledge regarding the possibilities of improving reaction time at the level of manual and foot exercises by practicing a specific exercise program that uses modern information technologies.

In all four tests specific to our study, statistically significant progress was recorded between the final and initial tests, thus demonstrating that the exercise program implemented using Fitlight technology was effective in optimizing reaction time at the level of manual and foot exercises of the pupils. Comparing the recorded progress of the male and female samples, we find that the female sample recorded superior progress, in the case of manual reaction time tests for all tests and for executions with the right and left hand, while the male sample excelled in the tests regarding the reaction speed involving the footwork, both for the right leg and for the left, in both tests with the spots arranged in a line (four Fitlights) and in a square (eight Fitlights). Analyzing the results, we find that the specific exercise program implemented in the study was effective. The differences recorded regarding the reaction time at the level of the executions that targeted manual and foot laterality on the right and left side, we consider to be due to the particularities of gender and motor experience.

The results of our study highlight the fact that the female sample recorded superior progress in terms of optimizing reaction time at the level of manual executions compared to the male sample that recorded better progress at the level of foot executions, regarding the manifestation of laterality and human reactions. The results of our studies contribute to confirming the conclusions of previous studies that aimed at the manifestation of different types of human reactions depending on the level of manifestation of laterality [52,53,54,55]. Moreover, the results of our study highlight aspects that were previously studied and through which highlighted gender differences regarding reaction time between male and female samples [55,56]. Several studies have focused on identifying the factors that influence human reaction time [48,57,58]; the correlation between the typology of physical activities, the level of motor capacity, and the possibilities of developing reaction time [59,60]; and gender differences in the manifestation of human reactions in physical activities and different sports [61,62]. Moreover, our study is in line with the studies that follow the development possibilities of the reaction time in relation to the particularities of age and gender [63,64].

Cerebral asymmetries in the processing and manifestation of the reaction time were numerous and followed the way in which the modal laterality manifests itself in relation to different stimuli: visual [65,66]; auditory [67,68]; and kinesthetic or combined [69,70,71]. Moreover, the same as in our study, previous studies have highlighted that the right and left manual or foot laterality is influenced by hemispheric asymmetry [72,73,74,75], by the speed of transmission of nerve impulses, by the ability to process simple or complex stimuli, by the time response, and by the size of the muscle segments or groups involved in the motor act [76,77,78]. We consider that the different results in the manifestation of reaction time in relation to manual and foot laterality are also due to preferences regarding motor activities; the samples of girls at the analyzed age prefer physical activities that predominantly involve manual laterality (volleyball, basketball, handball, dynamic games), while samples of boys prefer physical activities that involve executions in relation to the foot laterality, such as the game of football and other dynamic games aimed at foot actions [79,80]. Previous studies have highlighted gender preferences regarding the practice of the aforementioned types of activities and the physical level of the children, in which an important aspect analyzed concerned the level of manifestation of manual and/or foot laterality [81,82,83,84]. Our study differs from other studies by the fact that it tests students aged between 10 and 11 years old and aims to identify gender differences in manual and foot laterality by using Fitlight technology in the process of preparation and the testing of reactive time to visual stimuli. There are relatively few studies that have focused on the implementation of Fitlight technology in physical education lessons or physical activities for children, and mainly aim at the preparation and measurement of parameters of reaction time, agility, and hand-eye or foot-eye coordination in relation to the specifics of some motor and sports skills [26,27].

The strengths of our study were: the design and implementation of a specific exercise program to optimize reaction time through the use of modern information technologies, the large number of pupils included in the study, the analysis of gender differences in the manifestation of manual and foot reaction time, and the validation of some motor tests aimed at reaction time using Fitlight technology.

The limits of the research that can be identified consist in the relatively limited duration of the implementation of the specific program to optimize the reaction time based on the structure of the school year, the focus of the study only on a certain age sample, the exclusion of subjects with left manual and left foot laterality (due to the small number identified), and we did not target the dominant eyes in the analysis of the results of the study tests.

## 5. Conclusions

Our study highlights the fact that by implementing a specific exercise program which uses Fitlight technology, reaction time can be improved in relation to the manual and foot level of manifestation of laterality for both the right and the left side. By practicing the specific program that uses innovative technologies based on visual stimuli, the reaction time improved both at the level of manual and foot exercises. Through the comparative analysis of the samples and the process that aimed to optimize the reaction time specific to our study, it was found that the female sample recorded greater progress at the level of manual laterality, both for the right and for the left hand, while the boys’ sample had recorded significant progress in terms of improving the reaction time at the level of the right and left foot laterality. The results of the study show that the reaction time at the level of the right manual laterality was higher in both tests, so it was not influenced by the complexity of the test. At the foot laterality level, the results for the executions with the right foot were better in the simple test with four Fitlight spotlights in a line, and for the complex test with eight Fitlight spotlights in a square, the results were better in the executions with the left foot, which reveals the fact that the higher the complexity of the execution, the better the motor prevalence is on the left side.

## Figures and Tables

**Table 1:** Descriptive statistics of Fitlight test 4 spotlights in line—manual execution.

Fitlight 4 Spots in Line—Manual Execution									
Hand	Group	XTi ± SD	XTf ± SD	DX ± SD	95% CI		*t*	*p*	d
					Upper	Lower			
Right	Female	10.627 ± 2.040	11.986 ± 1.172	1.359 ± 0.426	1.536	0.419	3.242	0.009	0.644
	Male	10.727 ± 1.661	11.753 ± 1.712	1.025 ± 0.307	1.519	0.531	4.074	0.000	0.608
Left	Female	9.437 ± 1.365	10.193 ± 1.410	0.756 ± 0.762	1.507	0.604	4.591	0.000	0.760
	Male	9.334 ± 1.045	9.938 ± 1.064	0.604 ± 0.547	0.738	0.130	1.374	0.000	0.520

XTi—arithmetic average of initial test; XTf—arithmetic average of final test; SD—standard deviation; *t*—Student’s *t*-test; DX—average differences between final and initial tests; 95% CI—interval of confidence with lower and upper levels; *p*—statistically significant for *p* < 0.05; d—Cohen’s effect size.

**Table 2:** Descriptive statistics of the Fitlight test 4 spotlights in a line—foot execution.

Fitlight 4 Spots in a Line—Foot Execution									
Foot	Group	XTi ± SD	XTf ± SD	DX ± SD	95% CI		*t*	*p*	d
					Upper	Lower			
Right	Female	10.160 ± 1.687	10.979 ± 1.892	0.819 ± 0.115	0.996	0.358	2.699	0.002	0.456
	Male	10.245 ± 2.586	11.871 ± 2.137	1.625 ± 0.863	1.806	0.744	3.224	0.001	0.685
Left	Female	9.321 ± 2.366	10.650 ± 2.498	1.329 ± 0.346	1.670	0.510	3.901	0.000	0.546
	Male	9.417 ± 2.448	11.024 ± 2.832	1.607 ± 0.255	1.832	0.681	4.868	0.000	0.607

XTi—arithmetic average of initial test; XTf—arithmetic average of final test; SD—standard deviation; *t*—Student’s *t*-test; DX—average differences between final and initial tests; 95% CI—interval of confidence with lower and upper levels; *p*—statistically significant for *p* < 0.05; d—Cohen’s effect size.

**Table 3:** Descriptive statistics of the Fitlight test 8 spotlights in a square—manual execution.

Fitlight 8 Square Spotlights—Manual Execution									
Hand	Group	XTi ± SD	XTf ± SD	DX ± SD	95% CI		*t*	*p*	d
					Upper	Lower			
Right	Female	10.644 ± 2.133	11.827 ± 2.456	1.183 ± 0.280	1.437	0.672	2.293	0.012	0.514
	Male	10.524 ± 2.128	11.761 ± 2.095	1.080 ± 0.123	1.234	0.589	2.072	0.000	0.585
Left	Female	10.413 ± 2.154	11.491 ± 2.590	1.078 ± 0.887	1.317	0.738	2.103	0.036	0.452
	Male	10.451 ± 1.667	11.322 ± 2.024	0.871 ± 0.346	1.317	0.436	1.897	0.004	0.469

XTi—arithmetic average of initial test; XTf—arithmetic average of final test; SD—standard deviation; *t*—Student’s *t*-test; DX—average differences between final and initial tests; 95% CI—interval of confidence with lower and upper levels; *p*—statistically significant for *p* < 0.05; d—Cohen’s effect size.

**Table 4:** Descriptive statistics of the Fitlight test 8 spotlights in a square—foot execution.

Fitlight 8 Spots in a Square—Foot Execution									
Foot	Group	XTi ± SD	XTf ± SD	DX ± SD	95% CI		*t*	*p*	d
					Upper	Lower			
Right	Female	9.703 ± 1.686	10.454 ± 1.702	0.750 ± 0.569	0.886	0.286	3.576	0.015	0.443
	Male	9.801 ± 1.509	11.017 ± 2.012	1.216 ± 0.791	1.670	0.762	5.264	0.000	0.683
Left	Female	9.453 ± 1.498	10.112 ± 1.711	0.659 ± 0.477	0.152	0.563	3.169	0.011	0.409
	Male	9.711 ± 1.602	10.797 ± 2.103	1.086 ± 0.629	1.326	0.539	5.017	0.000	0.580

XTi—arithmetic average of initial test; XTf—arithmetic average of final test; SD—standard deviation; *t*—Student’s *t*-test; DX—average differences between final and initial tests; 95% CI—interval of confidence with lower and upper levels; *p*—statistically significant for *p* < 0.05; d—Cohen’s effect size.
